# Supplementary material for: (-)-Epigallocatechin-3-Gallate Prevents IL-1β-Induced uPAR Expression and Invasiveness via the Suppression of NF-κB and AP-1 in Human Bladder Cancer Cells
Source: Int J Mol Sci. 2022 Nov 13;23(22):14008. doi: 10.3390/ijms232214008 (PMC9697952; doi:10.3390/ijms232214008)
Supplement: Supplementary file 1 [file ijms-23-14008-s001.zip › ijms-2009177-supplementary.pdf]

Supplementary data and information

Article

(-)-Epigallocatechin-3-Gallate Prevents IL-1β-Induced uPAR Expression and Invasiveness via the Suppression of NF-κB and AP-1 in Human Bladder Cancer Cells

Dhiraj Kumar Sah 1, Pham Ngoc Khoi 2, Shinan Li 1, Archana Arjunan 1, Jae-Uk Jeong 3 and Young Do Jung 1,*

1 Department of Biochemistry, Chonnam National University Medical School, Hwasun 58128, Korea

2 Faculty of Basic Medical Sciences, Pham Ngoc Thach University of Medicine, Ho Chi Minh City 740500, Vietnam

3 Department of Radiation Oncology, Chonnam National University Medical School, Hwasun 58128, Korea

* Correspondence: ydjung@chonnam.ac.kr

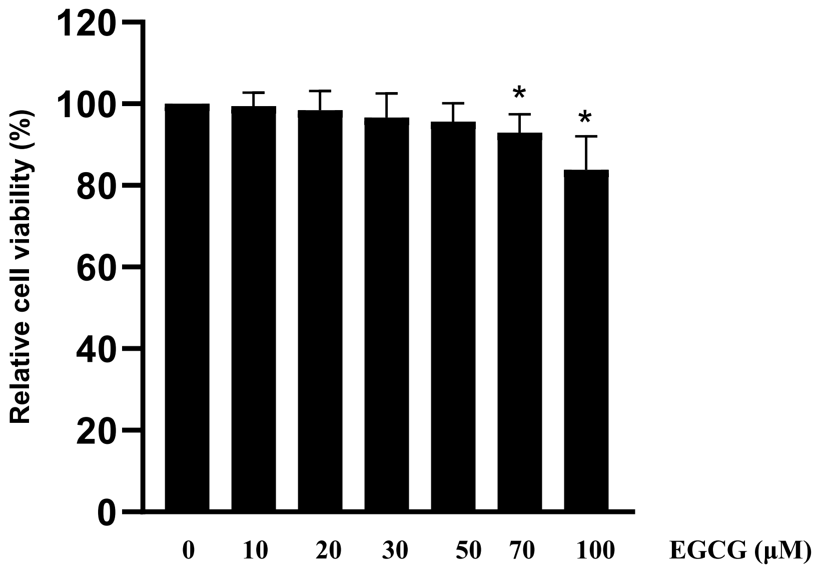

Supplementary Figure S1. EGCG MTT assay. T24 cells were incubated with 10-100 μM EGCG for 24 hours, and their viabilities were examined by performing the MTT assay. The above data represent the mean ± SD from triplicate measurements. * $p < 0.05$, versus control

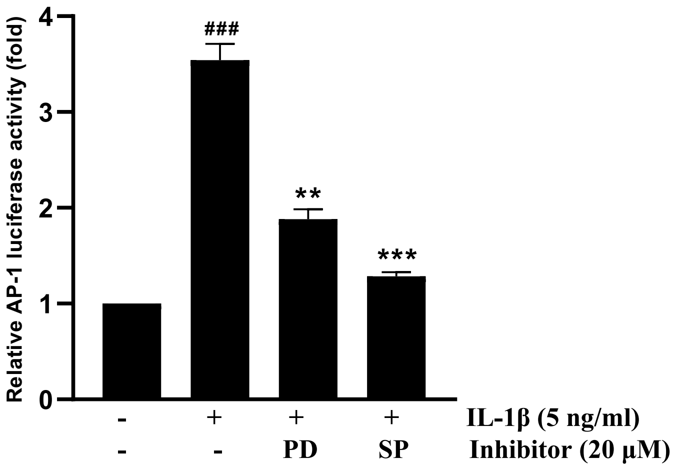

Supplementary Figure S2. Efficiency of specific chemical inhibitors of ERK1/2 (PD) and JNK (SP) signaling. T24 cells were pretreated with PD and SP for 1 hour and then were transiently transfected with the AP-1 luciferase reporter plasmid and were incubated with IL-1β, the cells were lysed, and luciferase activity was determined. The above data represent the mean ± SD from triplicate measurements. (# $P < 0.05$, ## $P < 0.01$, ### $P < 0.001$, #### $P < 0.0001$ versus control; * $p < 0.05$, ** $P < 0.01$, *** $P < 0.001$, **** $P < 0.0001$ versus IL-1β).

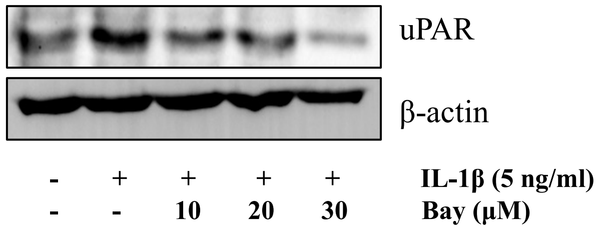

Supplementary Figure S3. T24 cells were pretreated with different concentration of Bay (10-30μM) for 1 hr. The cells were then incubated with IL-1β for 4 hr and uPAR protein level was evaluated by western blotting.

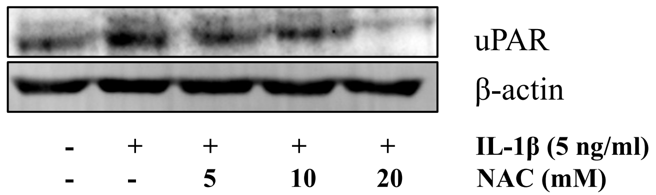

Supplementary Figure S4. T24 cells were pretreated with different concentration of NAC (5-20μM) for 1 hr. The cells were then incubated with IL-1β for 4 hr and uPAR protein level was evaluated by western blotting.
